# Changes in Appetite-Dependent Hormones and Body Composition After 8 Weeks of High-Intensity Interval Training and Vitamin D Supplementation in Sedentary Overweight Men

**DOI:** 10.3389/fnut.2022.827630

**Published:** 2022-02-07

**Authors:** Dariush Sheikholeslami-Vatani, Naser Rostamzadeh

**Affiliations:** Department of Physical Education and Sport Sciences, University of Kurdistan, Sanandaj, Iran

**Keywords:** HIIT, appetite, vitamin D3, acylated ghrelin, PYY

## Abstract

Exercise and diet are important factors for energy balance and appetite regulation. The aim of this study was to investigate the effect of 8 weeks High Intensity Interval Training (HIIT) and vitamin D_3_ supplementation in sedentary overweight men. Forty-eight participants were randomly assigned to one of the following four groups (*n* = 12): HIIT + VitD, HIIT + placebo (3 sessions per week, 10 × 1 min interval cycling at 90–100% VO_2peak_ separated by 1 min active recovery at 15% VO_2peak_for 8 weeks), Vit D and control groups. Participants received 2,000 IU/day 25 (OH) D_3_ or placebo. Measurements were taken pre and post training after 10 h overnight fasting. Insulin, weight, BMI and body fat percentage were significantly decreased, but PYY was significantly increased in the HIIT + Vit D and HIIT + placebo groups (*p* = 0.001 and *p* = 0.001, respectively) after 8 weeks of HIIT. Insulin (*p* = 0.009, *p* = 0.001), weight, BMI and body fat percentage (*p* = 0.001, *p* = 0.001) were significantly lower in the HIIT + Vit D and HIIT + placebo groups compared to the Vit D and control groups. However, PYY was significantly higher in the HIIT + Vit D group compared to the Vit D (*p* = 0.025) and control groups (*p* = 0.007) and also in the HIIT + placebo group compared to the Vit D (*p* = 0.037) and control groups (*p* = 0.032) after 8 weeks of HIIT. The combination of regular HIIT with vitamin D supplementation has a effect on appetite control and body composition.

## Introduction

Today, controlling and preventing weight gain is one of the most important factors in preventing disease and mortality in the world (1). Control of appetite and energy intake is a complex issue and depend on a variety of hormonal-neurological, psychological and even cultural factors. At the physiological level, appetite regulating gut hormones play an important role in hunger and satiety (2). PYY is an anorexigenic peptide, synthesized from L-cells in the gastrointestinal tract and released into the bloodstream. In this connection, acylated ghrelin an orexigenic gut peptide, is also released in the stomach (3). It is now well-known that exercise reduces orexigenic peptide (acylated ghrelin) and increases anorexigenic peptides (i.e., PYY) (4, 5). Results of many studies have shown a link between physical activity and physiological mechanisms of appetite control (4–6). As inactivity increases, a positive energy balance and subsequent weight gain occurs. However, physical activity plays a central role in the management of body weight by creating a negative energy balance and affecting the sensitivity of appetite-regulating hormones (7). One of the most essential aspects of exercise that might affect appetite regulation is the intensity of exercise (8).

The American college of sports medicine (ACSM) recently stated that high-intensity interval training (HIIT) is as effective as moderate-intensity continuous training (MICT) in improving body composition and insulin sensitivity in overweight and obese people (9). In this regard, high-intensity interval training\which involves short repetitive periods of activity with an intensity >85% of VO_2max_ has been shown to have similar and in some cases more physiological and metabolic adaptations in comparison with moderate-intensity endurance work (10). Recent evidence suggests that HIIT alters appetite-regulating hormones, limits energy consumption (8) and leads to a significant reduction in body fat mass (11).

On the other hand, Vitamin D deficiency is a global public health problem (12). According to the some estimations, more than one billion people in the world suffer from a deficiency of this vitamin (13). Some studies have shown that there is a significant deficiency of vitamin D_3_ in the adult population of different countries (35% in the United State, over 80% in Pakistan and Bangladesh, 90% in Turkey, 96% in India, and 67% in Iran) (13, 14). An inverse relationship has been found between the concentrations of 25 (OH) D and body fat mass (15). Excessive fat accumulation causes enzymatic disorders such as decreased activity of alpha-hydroxylase, the key enzyme in the conversion of 25-hydroxyvitamin D_3_ to 1,25-dihydroxyvitamin D_3_. This causes the accumulation of inactive forms and decreased bioavailability of vitamin D (16). As mentioned, vitamin D deficiency is associated with genesis of overweight and obesity (17). Reasons for decreased vitamin D levels during obesity include increased absorption of vitamin D by adipose tissue, decreased liver synthesis of vitamin D due to hepatosteatosis, and increased clearance of vitamin D during inflammation conditions (18, 19). In addition, vitamin D insufficiency increases parathyroid hormone concentration, activated lipogenesis, and results in greater accumulation of fat mass (20, 21). Vitamin D deficiency is associated with obesity and it has been reported that Vitamin D supplementation has similar effects of exercise on glucose metabolism and insulin sensitivity in overweight and obese individuals (22, 23).

It is believed that the hypothalamus will increase appetite and reduce energy expenditure due to low circulating levels of calcidiol due to vitamin D deficiency. These adjustments are made through the transcription pathways of the Neuropeptide Y (NPY) or Agouti related protein (AgRP) (24, 25). Vitamin D supplementation increases the expression of vitamin D receptor (VDR) gene in the pancreas (26), and VDR activation induces peptide YY transcription in pancreatic islets (an appetite suppression hormone that is produced in the pancreas in addition to intestinal L cells) (27). Daily intake of vitamin D supplements can maintain a sufficient serum level (above 30 ng/ml), but the daily dose varies according to age, gender, geographical location, skin pigmentation, physical activity and season. Research studies have illustrated that 2,000 IU/day is the minimum dose required to ensure a minimum goal (30 ng/ml) in the blood (28).

Independently, exercise and vitamin D_3_ supplementation both directly and indirectly induce beneficial and adaptive responses to control obesity, appetite, and body fat loss. However, there is a lack of research on the simultaneous effect of vitamin D_3_ supplementation and exercise on these variables. Therefore, the purpose of the present study was to investigate the simultaneous effect of HIIT and vitamin D_3_ supplementation on appetite-dependent hormones and body composition in overweight sedentary men.

## Materials and Methods

### Subjects and Experimental Design

Forty-eight Healthy and Overweight Male College Students (age: 21.7 ± 1.4 yr, Height: 175.3 ± 4.3 cm, Weight: 86.52 ± 3.92 kg and BMI: 27.28 ± 0.76 kg·m^−2^) Were Recruited From the University of Kurdistan to Participate in This Randomized Control Trial Study. Participants Were Randomly Assigned to four Groups: HIIT + Vitamin D, HIIT + Placebo, Vitamin D and Control Group. The Demographic Characteristics of the Subjects can be Seen in Table 1. The Sample Size Calculation Was Carried out Using G Powers Software (Heinrich-Heine-Universität, Düsseldorf, Germany). Questionnaires Covering Health History, Drug and Dietary Supplement Usage (3 months Prior to Study), Lacking Regular Exercise Training (in the Last 6 months) were completed by all subjects to determine eligibility. Having a Body Mass Index (BMI) Higher than 25 kg/m^2^ and Vitamin D Deficiency (20 ng/ml) were the Inclusion Criteria and all Subjects Had Vitamin D Deficiency Before the Intervention (Table 1).

**Table 1:** Demographic characteristics of the participants.

**Variable**	**Group**	**M** **± SD**		***P*-value**
		**Pre-intervention**	**Post-intervention**	
Height (cm)	HIIT + Vit D	178 ± 3.92		
	HIIT + Placebo	177.9 ± 4.42		
	Vit D	177.5 ± 4.1		
	Control	177.3 ± 3.95		
Weight (kg)	HIIT + Vit D	87.25 ± 3.36	*α82.10 ± 2.91	¶ 0.001
	HIIT + Placebo	86.25 ± 2.93	*α82.40 ± 2.66	¶ 0.001
	Vit D	86.11 ± 2.92	86.02 ± 2.88	0.41
	Control	86.06 ± 3.19	86.11 ± 3.21	0.42
BMI (kg/m^2^)	HIIT + Vit D	27.23 ± 0.36	*α25.48 ± 0.40	¶ 0.001
	HIIT + Placebo	27.25 ± 0.31	*α25.85 ± 0.44	¶ 0.001
	Vit D	27.32 ± 0.26	27.26 ± 0.25	
	Control	27.36 ± 0.35	27.37 ± 0.34	
BF (%)	HIIT + Vit D	35.18 ± 1.83	*α30.51 ± 1.88	¶ 0.001
	HIIT + Placebo	35.24 ± 1.74	*α31.08 ± 1.64	¶ 0.001
	Vit D	35.32 ± 1.63	35.17 ± 1.62	0.35
	Control	35.39 ± 1.65	35.41 ± 1.65	0.46
Vitamin D (ng/ml)	HIIT+ Vit D	16.1 ± 1.01	*€34.6 ± 2.1	¶ 0.001
	HIIT+ Placebo	15.86 ± 1.05	15.91 ± 0.98	0.35
	Vit D	15.99 ± 0.89	*€33.9 ± 2.47	¶ 0.001
	Control	15.78 ± 0.96	15.77 ± 1.03	0.65

HIIT, High-intensity interval training group; Vit D, Vitamin D group; BMI, Body mass index; BF, Body Fat percent; **¶**Significant difference compared to the pre-test; *Significant difference compared to the control group; **α**Significant difference compared to the Vitamin D group; €Significant difference compared to the HIIT+Placebo group.

They were informed about the associated risks and potential benefits of participation before giving their written consent. This study was approved by the Ethical Review Board of the University of Kurdistan (IR. UOK.REC.1398.024) and was conducted in accordance with the principles stated in the Declaration of Helsinki. The study design was registered at the registry of clinical trials and assigned the following number: IRCT2017050917675N2.

A familiarization session was carried out in order to familiarize participants with HIIT protocol and study procedures. During this session high, body weight and BMI of each participant was assessed and body fat percentage (BF) was also measured using the Jackson and Pollock equation and SAEHAN calipers (made in South Korea) (29).

### Dietary Records

In the present study, students living in the university dormitories with the same meals were used to relative control the nutritional status of the subjects. The daily meals of the university included three main meals of breakfast, lunch and dinner that ate at the central dining hall (without being able to choose different foods). Because it was possible for some subjects to consume other foods outside the university's diet program, the 24-h dietary recall form was used before the start of each blood sampling stages. The calorie intake from the four groups in the pre and posttest was analyzed by nutrition software (Table 2).

**Table 2:** The mean calorie intake of the subjects in the HIIT + Vitamin D, HIIT + Placebo, Vitamin D, and Control groups.

**Variable**	**Group**	**Pre-intervention**	**Post-intervention**	**Within-group *p*-value**
CHO (kcal)	HIIT + Vit D	*1, 384*±47	*1, 360*±45*α	0.001 ¶
	HIIT + Placebo	*1, 385*±48	*1, 363*±40*α	0.001 ¶
	Vit D	*1, 382*±50	*1, 388*±42	0.623
	Control	*1, 388*±46	*1, 387*±48	0.825
Fat (kcal)	HIIT + Vit D	485 ± 40	415 ± 35*α	0.001 ¶
	HIIT + Placebo	483 ± 38	415 ± 35*α	0.001 ¶
	Vit D	488 ± 42	485 ± 40	0.535
	Control	482 ± 40	484 ± 38	0.750
Protein (kcal)	HIIT + Vit D	265 ± 12	300 ± 12*α	0.001 ¶
	HIIT + Placebo	264 ± 14	301 ± 14α	0.001 ¶
	Vit D	266 ± 10	268 ± 10	0.812
	Control	265 ± 12	267 ± 12	0.835
Total calorie (kcal)	HIIT + Vit D	*2, 134*±50	*2, 075*±38*α	0.001 ¶
	HIIT+ Placebo	*2, 132*±55	*2, 079*±42*α	0.001 ¶
	Vit D	*2, 136*±48	*2, 141*±45	0.125
	Control	*2, 135*±52	*2, 136*±55	0.536

Data are presented as means ± SD. Vit D, Vitamin D group; CHO, carbohydrates; HIIT, High-intensity interval training group; ¶Significant difference compared to the pre-test; *Significant difference compared to the control group; αSignificant difference compared to the Vitamin D group.

In addition, mental perception of appetite (desire to eat and fullness) 24 h before the start of the training session and 48 h after the last training session was assessed in the fasting state using the Visual Analog Scale (VAS) in a continuum of zero to 100 Score (30) (Tables 3, 4).

**Table 3:** Appetite assess using visual analog scale (VAS).

**Variable**	**Group**	**M** **± SD**		**Within-group *p*-value**
		**Pre-intervention**	**Post-intervention**	
Hunger (mmVAS)	HIIT + Vit D	40.8 ± 10.5	27.4 ± 8.64	0.001^*^
	HIIT + Placebo	41.1 ± 10.9	26.9 ± 9.15	0.001^*^
	Vit D	41.5 ± 11	40.9 ± 11.85	0.655
	Control	40.7 ± 11.25	40.2 ± 11.5	0.718
Desire to eat (mmVAS)	HIIT + Vit D	42.5 ± 12.17	25.5 ± 7.64	0.001^*^
	HIIT + Placebo	41.9 ± 11.90	26.5 ± 7.15	0.001^*^
	Vit D	42 ± 13	41.7 ± 11.85	0.735
	Control	42.8 ± 12.25	42.1 ± 11.5	0.412
Satiety (mmVAS)	HIIT + Vit D	59.2 ± 11.2	72.6 ± 14.5	0.001^*^
	HIIT + Placebo	58.9 ± 11	73.1 ± 15.8	0.001^*^
	Vit D	58.5 ± 11.5	59.1 ± 14.5	0.752
	Control	59.3 ± 11.15	59.8 ± 14.17	0.812
Fullness (mmVAS)	HIIT + Vit D	57.5 ± 14.15	74.5 ± 16.65	0.001^*^
	HIIT + Placebo	58.1 ± 14.30	73.5 ± 16.5	0.001^*^
	Vit D	58 ± 13.90	58.3 ± 14.5	0.823
	Control	57.2 ± 14	57.9 ± 14.17	0.593

* *Significant difference at the level of p < 0.05. Vit D, Vitamin D group; HIIT, High-intensity interval training group*.

**Table 4:** Comparison of appetite between groups after the interventions.

**Variable**	**Post intervention**	***P***
Hunger (mmVAS)	HIIT + Vit D vs. HIIT+ Placebo	0.064
	HIIT + Vit D vs. Vit D	0.001^*^
	HIIT + Vit D vs. Control	0.001^*^
	HIIT + Placebo vs. Vit D	0.001^*^
	HIIT + Placebo vs. Control	0.001^*^
	Vit D vs. Control	0.63
Desire to eat (mmVAS)	HIIT + Vit D vs. HIIT+ Placebo	0.065
	HIIT + Vit D vs. Vit D	0.001^*^
	HIIT + Vit D vs. Control	0.001^*^
	HIIT + Placebo vs. Vit D	0.001^*^
	HIIT + Placebo vs. Control	0.001^*^
	Vit D vs. Control	0.72
Satiety (mmVAS)	HIIT + Vit D vs. HIIT + Placebo	0.078
	HIIT + Vit D vs. Vit D	0.001^*^
	HIIT + Vit D vs. Control	0.001^*^
	HIIT + Placebo vs. Vit D	0.001^*^
	HIIT + Placebo vs. Control	0.001^*^
	Vit D vs. Control	0.71
Fullness (mmVAS)	HIIT + Vit D vs. HIIT+ Placebo	0.75
	HIIT + Vit D vs. Vit D	0.001^*^
	HIIT + Vit D vs. Control	0.001^*^
	HIIT + Placebo vs. Vit D	0.001^*^
	HIIT + Placebo vs. Control	0.001^*^
	Vit D vs. Control	0.55

*Values are mean ± SD. Vit D, Vitamin D group; HIIT, High-intensity interval training group*.

* *Significant difference at the level of p < 0.05*.

### Training and Supplementation Protocols

A familiarization session was used to help participants understand how to carry out training protocol a week before the start of the training protocol at exercise physiology lab in university of kurdistan. During this session, body composition indices including height, body weight, body mass index, and body fat percentage was also measured. The training program was conducted entirely under the supervision of a member of research team. The experimental groups (HIIT + Vit D and HIIT + placebo groups) performed their HIIT program for 8 weeks, 3 sessions per week and each session lasted ~40 min. The exercise protocol involved 10 min warm-up, and 30 min main HIIT phase (10 × 1 min intervals cycling at 90% VO_2peak_ separated by 1 min active recovery at 15% VO_2peak_ for the first to fourth weeks and 10 × 1 min intervals cycling at 100% VO_2peak_ separated by 1 min active recovery at 15% VO_2peak_ for the fifth to eighth weeks) followed by 10 min cool-down (or recovery) in each session (31). The maximum heart rate was calculated by the Caronen formula and polar heart rate monitor model RS 400 (made in Finland) was used to control heart rate. The Vit D and control groups did not have any regular training program throughout the study period. HIIT + Vit D and Vit D group received 2,000 IU/day Vitamin D_3_ supplementation in capsule form and the HIIT + placebo group received placebo (Maltodextrin) capsules daily (28). Due to the fact that the subjects in the placebo group were deficient in vitamin D, at the end of the research protocol, they were also supplemented with vitamin D for 1 month according to the groups receiving the supplements.

### Blood Sample Analyses

In the first blood sampling (baseline), 8 ml of blood was drawn from a cubital vein under fasting conditions (10 h overnight fasting) at 8 am and the second blood sample was drawn 48 h after the last training session under the same conditions. Blood samples (8 mL) were transferred into tubes containing EDTA and a protease inhibitor [4-(2-aminoethyl) benzenesulfonyl fluoride hydrochloride (AEBSF)] to prevent the degradation of acylated ghrelin. The tubes containing EDTA were immediately centrifuged at 3,000 rpm at 4 C for 10 min. Then, samples were pipetted into micro tubes and immediately frozen at −80 C for later analysis. The acylated ghrelin, PYY and insulin plasma levels were determined using commercial kits (Human ELISA, HANGZHOU EASTBIOPHARM Co., LTD, CHINA) according to the manufacturer's protocol with a lower detection limit of 2.6, 2.53 ng/ml, and 0.25 μIU/ml, respectively. The intra-assay and inter-assay coefficients of variation were <10% and <12% for acylated ghrelin, <10% and <12% for PYY, and <6.45% and <6.45% for insulin, respectively. Fasting blood glucose (FBG) samples were taken in a sitting position following 10 h overnight fasting before and after the intervention. FBG was measured by biochemical autoanalyzer A15 with Biosystem kit (made by Spain).

### Statistical Analyses

Results are expressed as Mean ± SD. The normal distribution of data and homogeneity of variances were assessed using the Shapiro-Wilk and Levene tests, respectively. Then, dependent *t*-test was used to analyze within-group changes. Analysis of variance with repeated measure and Bonferroni *post-hoc* test were used to evaluate within-group, between-group, and interaction (time x group) (Table 5). Data was analyzed using SPSS for Windows version 23 (IBM Corp., Armonk, N.Y., USA). The significance level was set at *P* ≤ 0.05.

**Table 5:** The ANOVA test results regarding research variables.

**Variable**	**ANOVA**	***F***	***P***
Weight (kg)	Time	975.77	*0.001
	group × time	332.89	*0.001
	Group	58.12	*0.001
BMI (kg/m^2^)	Time	560.33	*0.001
	group × time	181.49	*0.001
	Group	60.31	*0.001
BF (%)	Time	932.3	*0.001
	group × time	412.28	*0.001
	Group	46.69	*0.001
Accylated ghrelin (pg/ml)	Time	1.66	0.203
	group × time	1.004	0.40
	Group	0.30	0.99
PYY (pg/ml)	Time	95.34	*0.001
	group × time	36.56	*0.001
	Group	6.12	*0.001
Insulin (ng/dl)	Time	339.28	*0.001
	group × time	110.53	*0.001
	Group	4.04	*0.013
Vitamin D (ng/ml)	Time	720.7	*0.001
	group × time	635.2	*0.001
	Group	84.89	*0.013
Glucose (mg/dl)	Time	1.02	0.283
	group × time	1.13	0.345
	Group	1.42	0.243

BMI, Body mass index; BF, Body Fat percent; PYY, Peptide YY. *Significant difference at the level of p < 0.05.

## Results

Prior to exercise intervention, no intergroup differences were observed in any of the study variables (acylated ghrelin, PYY, insulin, appetite, weight, BMI, body fat percentage, and plasma vitamin D level) (*p* > 0.05).

### Serum Concentrations of Acylated Ghrelin, PYY, Insulin, Glucose, and Vitamin D

**Figure 1:**
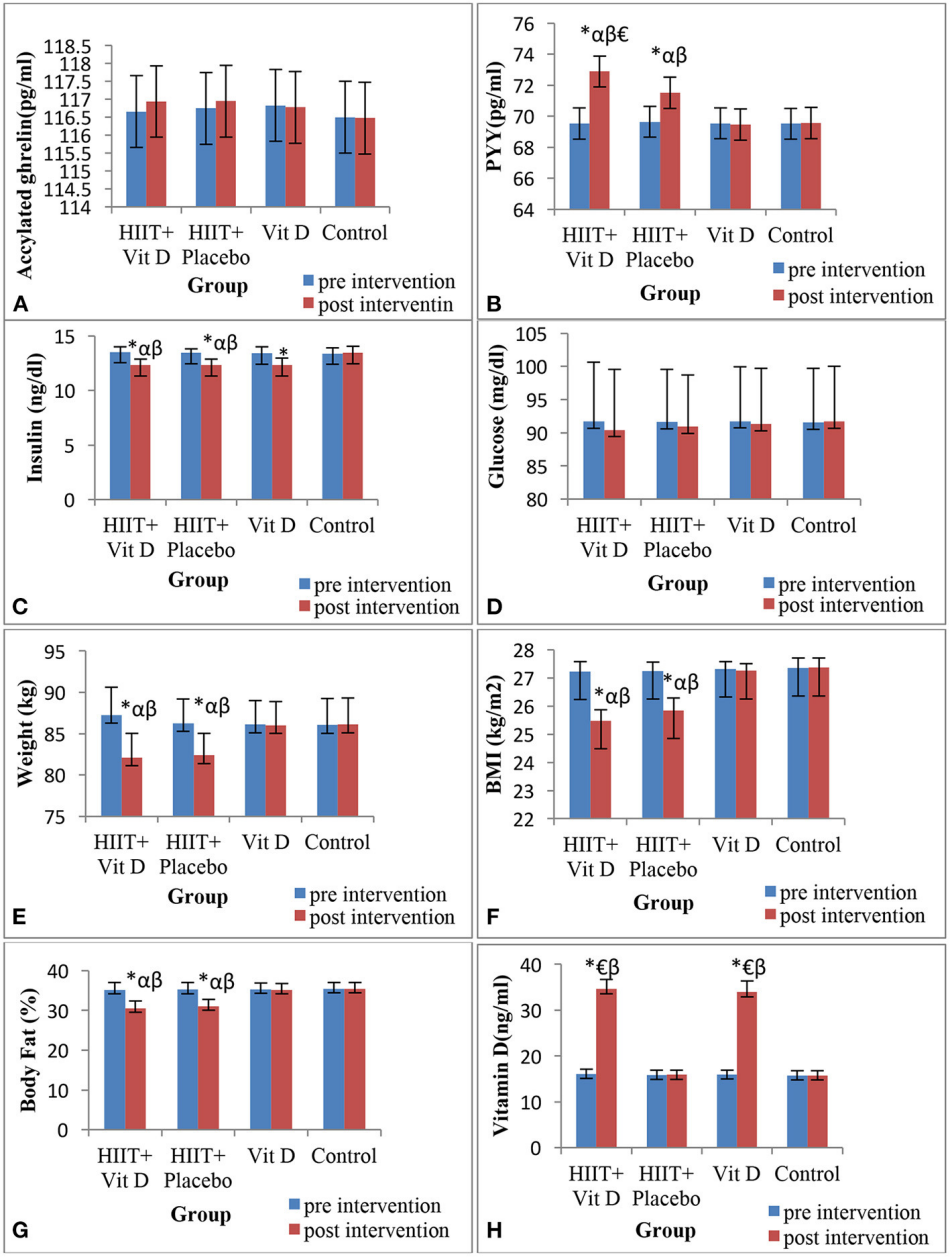
Changes in acylated ghrelin **(A)**, peptide YY (PYY) **(B)**, insulin **(C)**, glucose **(D)**, body weight **(E)**, BMI **(F)**, body fat percentage **(G)** and vitamin D **(H)** during 8 weeks of HIIT protocol in overweight sedentary men. *Significant difference compared to the pre-test; αSignificant difference compared to the Vitamin D group; βSignificant difference compared to the control group; €Significant difference compared to the HIIT + Placebo group.

Circulating levels of acylated ghrelin (Figure 1A) and glucose (Figure 1D) were not affected by any of the HIIT or vitamin D supplementation.

For the PYY hormone, there was a significant group × time interaction (*F* = 36.5, *P* = 0.001). The present results illustrated that PYY increased only in HIIT + Vit D (*P* = 0.001) and HIIT + placebo (*P* = 0.001) groups after 8 weeks of intervention. It was also revealed that PYY was higher in the post-intervention in HIIT + Vit D group compared to Vit D (*P* = 0.025), control (*P* = 0.007) and HIIT + placebo (*P* = 0.036) groups. PYY was also higher in the post-test in HIIT + placebo group compared to Vit D (*P* = 0.037) and control (*P* = 0.032) groups (Figure 1B).

There was a significant group × time interaction for insulin (*F* = 110.53, *P* = 0.001). Regarding this variable it was observed that insulin decreased in HIIT + Vit D (*P* = 0.001), HIIT + placebo (*P* = 0.001) and Vit D (*P* = 0.043) groups after interventions. In addition, it was demonstrated that insulin was lower in the post-intervention in the HIIT + Vit D group compared to Vit D (*P* = 0.001) and control (*P* = 0.001) groups. Furthermore, insulin was noticeably lower in the post-test in HIIT + placebo group compared to Vit D (*P* = 0.001) and control (*P* = 0.001) groups (Figure 1C).

There was significant group × time interaction (*F* = 635.2, *P* = 0.001) for serum concentrations of Vitamin D. In connection with this, it was shown that serum concentrations of Vitamin D increased in HIIT + Vit D (*P* = 0.001) and Vit D (*P* = 0.001) groups after 8 weeks. Serum concentrations of Vitamin D in HIIT + Vit D group compared to HIIT + placebo (*P* = 0.001) and control (*P* = 0.001) groups were higher. In addition, serum concentrations of Vitamin D were also higher in the post-intervention in Vit D group compared to HIIT + placebo (*P* = 0.001) and control (*P* = 0.001) groups (Figure 1H).

### Appetite Ratings (Hunger, Desire to eat, Satiety and Fullness) and Total Calorie Intake

There was a significant group × time interaction for total calorie intake (*F* = 10.86, *P* = 0.001). Current results showed that total calorie intake decreased in HIIT + Vit D (*P* = 0.001) and HIIT + placebo (*P* = 0.001) groups after 8 weeks (Table 2). Aftermore, total calorie intake in HIIT + Vit D and HIIT + placebo groups compared to Vit D (*P* = 0.001) and control (*P* = 0.001) groups were lower in the post-intervention (Table 2).

Findings on appetite ratings indicated that there were significant group × time interaction for hunger (*F* = 323.55, *P* = 0.001), desire to eat (*F* = 347.34, *P* = 0.001), satiety (*F* = 313.27, *P* = 0.001) and fullness (*F* = 267.44, *P* = 0.001). Ratings of hunger and desire to eat decreased in HIIT + Vit D (*P* = 0.001) and HIIT + placebo (*P* = 0.001) groups after 8 weeks of intervention (Table 3). Ratings of hunger and desire to eat in HIIT + Vit D group compared to Vit D (*P* = 0.001) and control (*P* = 0.001) groups, as well as in the HIIT + placebo group compared to Vit D (*P* = 0.001) and control (*P* = 0.001) groups were lower in the post-intervention (Table 4). Ratings of satiety and fullness increased in HIIT + Vit D (*P* = 0.001) and HIIT + placebo (*P* = 0.001) groups after 8 weeks (Table 3). It is confirmed that ratings of satiety and fullness in HIIT + Vit D group compared to Vit D (*P* = 0.001) and control (*P* = 0.001) groups, and in HIIT + placebo group compared to Vit D (*P* = 0.001) and control (*P* = 0.001) groups were notably higher in the post-intervention (Table 4).

### Body Weight, BMI and Body Fat Percent

There were significant group × time interaction for body weight (*F* = 332.89, *P* = 0.001), BMI (*F* = 181.49, *P* = 0.001) and body fat percentage (*F* = 412.28, *P* = 0.001) (Table 5). Body weight decreased in HIIT + Vit D (*P* = 0.001) and HIIT + placebo (*P* = 0.001) groups after 8 weeks of intervention. In the HIIT + Vit D group compared to Vit D (*P* = 0.012) and control (*P* = 0.010) groups body weight were lower in the post-intervention. The same result was observed for body weight in the HIIT + placebo group compared to Vit D (*P* = 0.025) and control (*P* = 0.020) groups (Figure 1E).

In regards BMI, it was decreased in HIIT + Vit D (*P* = 0.001) and HIIT + placebo (*P* = 0.001) groups after 8 weeks (Table 5). BMI in the HIIT + Vit D group compared to Vit D (*P* = 0.001) and control (*P* = 0.001) groups were lower in the post-test. A noticeable decrease in BMI was observed in the post-test for the HIIT + placebo group compared to Vit D (*P* = 0.001) and control (*P* = 0.001) groups (Figure 1F).

Body fat percentage also decreased in HIIT + Vit D (*P* = 0.001) and HIIT + placebo (*P* = 0.001) groups after 8 weeks of intervention (Table 5). In relation to this variable, it revealed that body fat percentage in the HIIT + Vit D group was greatly reduced compared to the Vit D (*P* = 0.001) and control (*P* = 0.001) groups in the post-intervention. The same result was obtained for the HIIT + placebo group compared to Vit D (*P* = 0.001) and control (*P* = 0.001) groups (Figure 1G).

## Discussion

The combination of exercise and diet affects energy balance and appetite regulation in individuals with obesity and is potentially a major mechanism in weight control. This study is the first to evaluate the effect of HIIT and vitamin D supplementation simultaneously on appetite, appetite-regulated hormones and body composition in sedentary overweight men.

The results of present study showed that 8 weeks of HIIT with an intake of 2,000 IU/day of vitamin D_3_ supplementation significantly reduced the serum levels of PYY and insulin, but no change was observed in the serum levels of acylated ghrelin. Numerous studies have evaluated the effect of HIIT on appetite-related hormones. In this regard, Liao et al. in a study proved that serum levels of orexin in people with obesity after 6 weeks of HIIT activity significantly decreased while acylated ghrelin did not change (32). Little et al. and Racil et al. also observed a significant decrease in serum insulin levels through an increase in GLUT4 on individuals with obesity after a 12 weeks of HIIT activity (33, 34). Although the reason for the change in appetite hormones after HIIT activity are still unknown, researchers believe that one of the reasons for these changes is due to the redistribution of blood flow from splanchnic areas to active skeletal muscle (35). Changes in insulin and glucose after HIIT might also be due to increased GLUT4 transporter protein, increased glycogen synthase and hexokinase activity in appetite and related hormones (36). Increased IL6 after exercise is one of the factors affecting appetite suppression (increased PYY) (37) which unfortunately was not evaluated in this study. On the other hand, Martins et al. showed that various exercises such as HIIT did not significantly change the acylated ghrelin and PYY of the subjects in the exercise groups (38). Larsen et al. also showed that HIIT reduced acylated ghrelin compared to moderate-intensity exercise in overweight sedentary men (39). Duration of exercise (one session vs. 8 weeks) as well as individual differences between subjects, including age and gender are likely reasons for the contradictory results (40). Sim et al. also did not observe a significant change in appetite-dependent hormones (acylated ghrelin and PYY) in individuals with overweight and obesity after 12 weeks of HIIT (8). The reason for the difference might be due to the training protocol, particularly the intensity of training.

The results of the present study demonstrated that overweight sedentary men, in addition to changes in appetite-dependent hormones experienced a significant decrease in weight, BMI, body fat percentage and mental perception of appetite after 8 weeks of HIIT activity. In confirmation of these results, Sim et al. showed that high-intensity exercise reduced the feeling of hunger and energy intake in overweight sedentary individuals up to 24 h after activity compared to moderate-intensity exercise (41). Liao et al. observed a significant reduction in body weight and BMI in individuals with obesity after 6 weeks of HIIT (32). Dupuit et al. also showed that HIIT reduced body fat percentage, body weight and BMI in women with overweight (42). One of the metabolic adaptations due to HIIT is an increase in fat oxidation by changing the metabolism pathway [decreasing the fatty acid synthase (FAS) enzyme and increasing fat oxidation] witch lead to changes in body composition (43). Weight loss might also be due to decreased energy intake and increased energy expenditure during exercise as recent evidence suggests that high-intensity activity could by regulating downstream signaling pathways of hunger hormones and upstream signaling pathways of satiety hormones (PYY) alter appetite (by altering appetite-regulating hormones, including PYY) and limit energy intake as an exercise-induced anorexia (41, 44). On the other hand, the findings of Larsen et al. illustrated that HIIT did not cause significant change in the subjects' appetite and energy intake compared to moderate-intensity exercise despite changes in appetite-dependent hormones (39). Changes in appetite- dependent hormones are not always consistent with the mental perception of appetite and the amount of calorie expenditure due to activity, and this indicates the complexity in regulating appetite and the impact of various physiological and psychological factors (45).

The results of the present study showed that vitamin D supplementation with HIIT caused significant changes in appetite-dependent hormones (PYY and insulin), body weight, BMI, body fat percentage and mental perception of appetite. Studies have also evaluated the effect of vitamin D supplementation on appetite-dependent hormones. In this regard, Bhatt et al. showed that taking vitamin D supplements in overweight women decreased insulin resistance and fasting blood glucose (FBG) (46). Vitamin D regulates insulin and increases insulin sensitivity directly and indirectly in pancreatic β cells (47). The direct effect of vitamin D on glucose metabolism might be through its binding to vitamin D receptors (VDR) in pancreatic β cells and activation of intracellular signaling pathways (47). Choi et al. showed that vitamin D indirectly increased the expression of the PYY hormone gene in the pancreas of mice. They showed that vitamin D supplementation increases the expression of vitamin D receptor genes in the pancreas and VDR activation induces peptide YY transcription in pancreatic islets (27). Regarding the effect of vitamin D supplementation on weight loss, BMI and body fat percentage, there is no research to show that vitamin D alone causes weight loss, decreased BMI and body fat percentage, but researchers have found a link between serum vitamin D levels and these variables. In this regard, Saliba et al. demonstrated that there is an inverse relationship between vitamin D supplementation with weight and BMI (48). Caron et al. also found that taking vitamin D supplements and reaching levels above 25 ng/dl was directly related to reducing body fat (49). Vitamin D can indirectly increase metabolism and reduce fat mass by increasing muscle mass, stimulating sympathetic nerves and reducing insulin (50). Vitamin D might also have a direct effect on adipogenesis and differentiation of fat cells and reduce the absorption of fatty acids in the intestine (51). Vitamin D increases fat oxidation by regulating genes involved in fatty acid oxidation and mitochondrial metabolism, thus limiting weight gain (52).

Overall, the findings of this study showed that HIIT cause changes in appetite-dependent hormones, decrease appetite, weight, body fat percentage and BMI, and if these exercises are accompanied with an intake of 2,000 IU/day of vitamin D_3_, changes in the variables are more perceptible.

## Data Availability Statement

The raw data supporting the conclusions of this article will be made available by the authors, without undue reservation.

## Ethics Statement

The studies involving human participants were reviewed and approved by Ethical Review Board of the University of Kurdistan (IR.UOK.REC.1398.024). The patients/participants provided their written informed consent to participate in this study.

## Author Contributions

DS-V performed the analytic calculations and supervised the project. All authors contributed to the article and approved the submitted version.

## Conflict of Interest

The authors declare that the research was conducted in the absence of any commercial or financial relationships that could be construed as a potential conflict of interest.

## Publisher's Note

All claims expressed in this article are solely those of the authors and do not necessarily represent those of their affiliated organizations, or those of the publisher, the editors and the reviewers. Any product that may be evaluated in this article, or claim that may be made by its manufacturer, is not guaranteed or endorsed by the publisher.
